# Clinical Assessment of the Congenital Absence of Palmaris Longus and Flexor Digitorum Superficialis Muscles in Young Saudi Population

**DOI:** 10.1155/2017/5342497

**Published:** 2017-04-12

**Authors:** Mohammed Talal Alzahrani, Mohammad Abdullah Almalki, Turki Abdullah Al-Thunayan, Amjaad Hamad Almohawis, Ahmed Turki Al Turki, Loung Umedani

**Affiliations:** ^1^King Saud Bin Abdulaziz University for Health Sciences (KSAU-HS), Riyadh, Saudi Arabia; ^2^Basic Medical Sciences Department, College of Medicine, King Saud Bin Abdulaziz University for Health Sciences, National Guard Health Affairs (NGHA), Mail Code 3127, P.O. Box 3660, Riyadh 11481, Saudi Arabia

## Abstract

*Introduction.* Congenital Palmaris Longus (PL) absence was found in 15%–20.25% of population globally. This condition and Flexor Digitorum Superficialis (FDS) tendon absence in little finger are not known in Saudi Arabia. We studied prevalence of PL and FDS agenesis in Saudi Arabian population.* Methods.* A random cross-sectional study was carried out after an ethical approval in the Riyadh universities. Schaeffer's test was used to examine PL absence. The Modified test was used to examine FDS absence. Data was analyzed using Microsoft Excel and the SPSS Software version 22.* Results.* The volunteers, 331, males 164 (49.5%) and females 167 (50.5%), mean age of 23 (SD ± 5.3), showed right hand dominance in 294 (88.8%) and bilateral absence of PL and FDS in 15.1% and 14.8%, respectively. The hand dominance showed no significant relation between PL and FDS absence, *p* value = 0.788, 0.835, respectively. Generally, we found a weak correlation between absence of the PL and FDS, *p* value ≥ 0.595.* Conclusion.* The bilateral absence of PL and FDS was found as 15.1% and 14.8%, respectively. Variation of the FDS tendon absence was an independent entity for the PL absence. The dominance of hands was not related to the tested variables found in PL and FDS agenesis.

## 1. Introduction

Palmaris Longus (PL) is a member of one of the four muscles which form the superficial layer of the anterior forearm. Although it is a phylogenetically degenerating muscle with a very short belly, yet it functions as a weak flexor of the wrist [1]. It is located between the flexor carpi ulnaris medially and the flexor carpi radialis laterally. It arises commonly with the other flexors from the medial epicondyle of the humerus and attaches to the flexor retinaculum where it is spread to mingle with the palmar aponeurosis [1]. The congenital absence of the PL muscle was initially reported in 1944 by Reimann et al. as 15% in the global population [2].

Numerous studies show variations in the percentages of PL muscle agenesis in different geographical regions [3]. A systematic review composed of 26 articles revealed the prevalence of PL absence by 20.25% globally. In the Arab Middle Eastern regions several studies revealed a significantly higher absence of PL (i.e., 41.7%) [3]. The lowest prevalence of PL absence was observed in black Africans in general [4]. Specifically, Uganda is the lowest (1.02%) followed by Zimbabwe (1.5%) [5, 6]. Absence of PL was 3.7% more in females in Iran [7]. The highest prevalence was found in Turkey (63.9%) [8]. In Saudi Arabia, few studies have been conducted for the estimation of PL muscle absence among different regions and ethnic groups. A cross-sectional study explored the prevalence of PL muscle absence in Jizan population in Saudi Arabia in which it was estimated to be 16.7%. This was considered within the prevalence of the global population [3, 9].

Flexor Digitorum Superficialis (FDS) is the only muscle of the intermediate layer of the forearm muscles. It arises by two heads, a humeroulnar head and a radial head. They arise from the medial epicondyle of the humerus and the anterior oblique line of the radius, respectively. FDS has four tendons that attach to the palmar aspect of the index, middle, ring, and little fingers. FDS is responsible for flexion of multiple joints crossed by its tendons, that is, wrist joints, intercarpal joints, carpometacarpal joints, metacarpophalangeal joints, and the proximal interphalangeal joints [1]. The prevalence of functional FDS muscle agenesis for the little finger in Saudi Arabia is unknown. In contrast, the prevalence of FDS absence in other studied populations is known, for example, in the Caucasian population (15–21%), in the Chinese population (6.4%), and in the Indian population (0.25%) [10, 11].

The PL is an accessory muscle with a lesser functional significance. The absence of the PL has a negligible effect on the strength of the handgrip [12–14]. Surgically, it has been used as an ideal tendon graft in a wide variety of procedures in plastic surgeries and in the procedures carried out on the hand itself [13, 14]. Similarly, the absence of the tendon of the FDS for the little finger does not affect the functionality of the little finger [15]. In addition, when the little finger is injured, some surgeons tend to examine the other hand to check for the absence of FDS assuming the agenesis is bilateral [16, 17]. Based on the above indicated observations, we studied the prevalence of the absence of both the Palmaris Longus and the Flexor Digitorum Superficialis muscles in the Saudi Arabia population.

## 2. Objectives

The objectives were as follows:

- To study the prevalence of congenital absence of Palmaris Longus muscle and the Flexor Digitorum Superficialis tendon in the Riyadh city universities.
- To compare the results of the studied prevalence with the national and international observations.

## 3. Materials and Methods

This study was carried out in the Saudi Arabian population on *N* = 331 volunteers, males 164 (49.5%) and females 167 (50.5%), in Riyadh city. This was a random cross-sectional study conducted from January 2016 after an approval from the Ethical Review Committee of the King Abdullah International Medical Research Center (KAIMRC). We carried out our study on the volunteers from four government based universities in Riyadh city. The total number of students (i.e., volunteers) present in these universities was 156,733. These were King Saud bin Abdulaziz University for Health Sciences (KSAU-HS) [18], college of medicine, 850 volunteers, King Saud University (KSU), 66,174 volunteers, Imam Muhammad ibn Saud Islamic University (IMAM), 37,401 volunteers [19], and Princess Nourah Bint Abdulrahman University (PNU), 52,308 volunteers [20]. We calculated the sample size (≈284 volunteers) for this population, that is, 156,733, with a 95% confidence interval level, ±5% margin of error, and estimated agenesis of 24.5% by using the Raosoft Inc. calculator [9]. We used the Cluster Sampling technique in the indicated universities, that is, PNU, KSAU-HS, KSU, and IMAM, and took the volunteers at random from this population. Randomization was done by dividing each university into colleges, and each college was divided into specialties and each specialty was divided into batches by academic year. Three colleges were chosen from each university by simple random sampling. In each college, we took three specialties by simple random sampling. In each specialty, we picked three batches randomly, and, from each batch, we selected three students by systematic random sampling via a computer generator randomization. So total sample size was 3 students per batch × 3 batches per specialty × 3 specialties by college × 3 colleges per university × 4 universities = 324 volunteers, but we selected 331 volunteers to attain the total number if someone shows noncompliance. We distributed entire population individuals to the coinvestigator's groups. In each of the groups the volunteers were asked to fill up a questionnaire and they signed an informed consent.

The volunteers were given information about the examination and protocols of the project they were about to participate in. The inclusion and the exclusion criteria were decided. In this study, we recruited any Saudi subject who was willing to voluntarily participate while studying at any academic level. Volunteers from both genders were enrolled. We excluded the volunteers who had a positive history of trauma on their hands and forearm, volunteers showing any sign of inflammation of the hands or forearms, and students with a history of surgical intervention on their hands and presence of any other known congenital anomaly of the hands.

During the first visit of the subject, the investigating medical interns (one female and one male) were fully educated and trained to carry out the clinical examination of the hand using tests to assess the PL and FDS absence. The accuracy of the methods used by the students to diagnose the agenesis was counterchecked by the PI who is a clinician as well as the subject expert. The collected data was entered in an Excel sheet with the subject's serial number, gender, nationality, date of birth, age, and the pattern of dominance of hand. The pattern of hand dominance was determined by history and observation by asking the patient to write on a paper.

**Figure 1:**
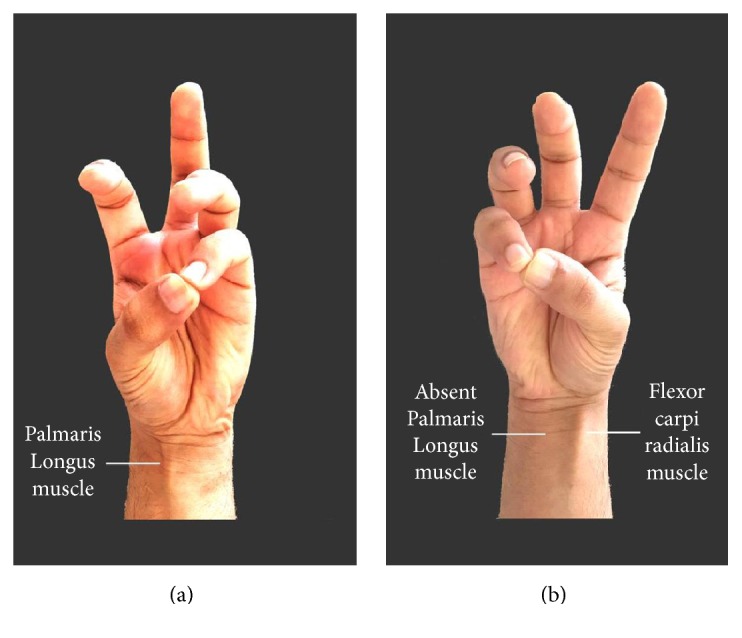
Volunteer demonstrating unilateral absence of Palmaris Longus (b).

**Figure 2:**
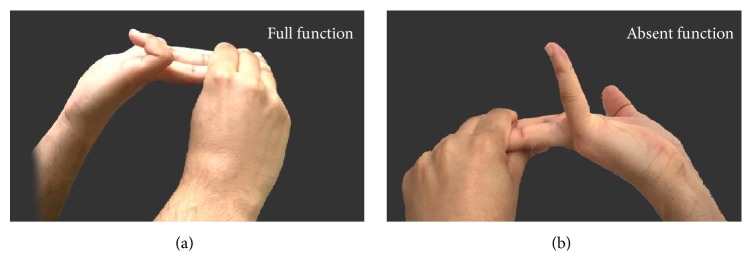
Volunteer demonstrating full function of the little finger FDS (a). Volunteer demonstrating absence of little finger FDS function (b).

Both hands of the participant were assessed for PL and FDS absence. The data were entered with the following mean parameters: (i) presence of anomalies in both hands, (ii) absence of anomaly in both hands, (iii) presence of anomaly in the right hand only, and (iv) presence in the left hand only. In order to test the absence of the PL, Schaeffer's test was used, in which the wrist was slightly flexed and the thumb and little finger were opposite each other. This caused a raised ridge just proximal to wrist which indicated the presence of the PL muscle and absence of this ridge was diagnostic of PL muscle absence (Figure 1) [10]. The FDS absence was tested by using a Modified test in which the subject was asked to flex the fourth and fifth digit while the interphalangeal joints of the other digits were held in full extension by the examiner's hand to prevent any effect generated by the flexor digitorum profundus tendon. Failure of the subject to flex the fourth or fifth digit is diagnostic of FDS absence. This test has a known high accuracy for FDS absence (Figure 2) [21].

The data was entered and coded in MS Excel and SPSS package (version 22) for the statistical analysis. Descriptive statistics for the prevalence of congenital agenesis of PL and FDS muscles in the study was calculated as frequencies and percentages. Chi-square test was used to assess the relation between FDS/PL agenesis versus gender/dominant hand and PL agenesis versus FDS agenesis. A *p* value of <0.05 was identified as statistically significant.

## 4. Results

A total of 331 volunteers were included in the study. It consisted of 164 (49.5%) males and 167 (50.5%) females. The mean age was observed as 23 (SD ± 5.3) years in this population (Table 1).

By questioning the participants, we found that 294 volunteers (88.8%) had right hand dominance as opposed to (11.2%) who had left hand dominance. The aspect of the dominance of the hands did not generate any significant result; that is, *p* value ≥ 0.05 (Tables 2 and 3).

Bilateral absence of PL was observed in 50 volunteers (15.1%) (Table 2). Bilateral absence of FDS was noted in 49 volunteers (14.8%) (Table 3). We observed a statistically significant difference between the status of absences of the PL and the FDS in the volunteers based on the gender distributions, *p* value ≤ 0.05 (Tables 2 and 3).

We observed no significant correlation in the variations of PL status and FDS status, *p* value = 0.595 (Table 4).

A Tukey post hoc test revealed that presence of FDS in the left hand was statistically significant with a female gender predominance, *p* value = 0.005. There was no statistically significant difference between the other variations.

## 5. Discussion

**Figure 3:**
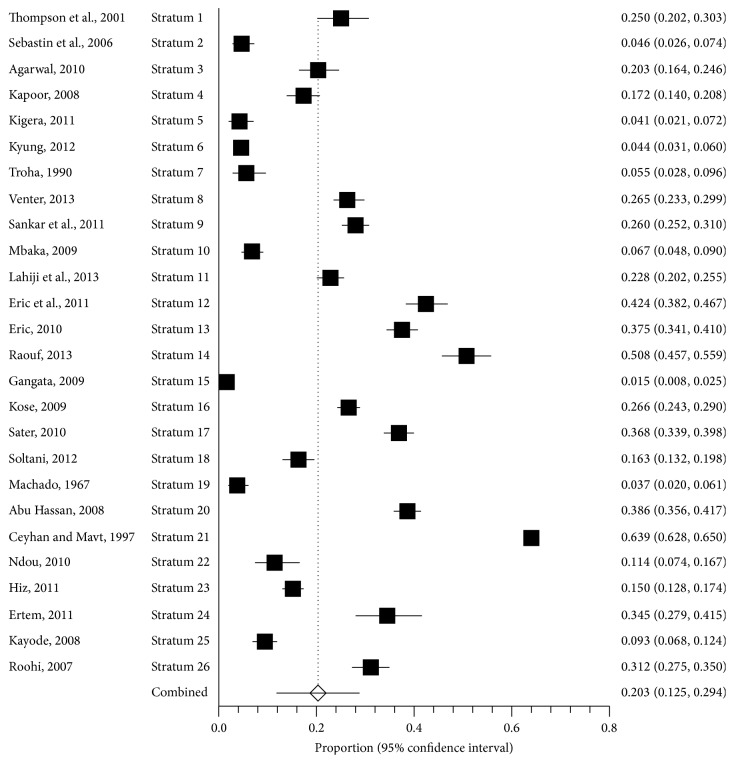
Global prevalence of the Palmaris Longus muscle as studied by Yammine showing overall PLA proportion meta-analysis plot.

In a review by Yammine, published in 2013, a higher prevalence of PL absence (i.e., 41.7%) was reported in the Arabian countries (Figure 3) [3]. The PL absence is strongly known to have a relation with racial variations; for example, the Caucasians population in general and the Turkish population specifically showed 26.3% and 34.1%, respectively [3]. On the contrary, in comparison our findings showed 15.1% absence of the Pl and it is a lesser percentage than what Yammine showed (i.e., it was a higher prevalence).

In some multiple studies carried in 2015 by Ioannis et al., a wide range of the percentage of the PL absence (1.5%–63.9%) was noted. It was also specified that the Caucasian population showed 5.5% prevalence [4]. In our study, the percentage lies within Ioannis range. Moreover, the results of such kind of studies which were conducted at the global level support our results. A study carried out by Lahiji et al., in 2013, showed increased prevalence of the PL absence in females by 3.7 times compared to males [7]. In our findings, the gender based distribution showed significant results. Many authors have reported a correlation between PL absence and the handedness. Generally, the right hand dominance was reported more in its prevalence than the left hand dominance [22]. Although it is a known fact, it was tested regarding the relation of the dominance with the simultaneous PL absence. It was noted in the studies that the PL absence was found to be more common in the right versus the left hand dominance population [23]. However, other researchers did not find any association between the hand dominance and PL absence. In our study, we could not identify any difference between handedness and this will not add any change in the available literature.

The relation of the effect of the PL absence with the function of the FDS was also observed in some studies. The FDS was associated usually with an absence of PL muscle. Thompson et al. reported the effects of PL absence causing decrease in power of the FDS action [24]. In an Indian study, significant results were obtained showing the FDS weakness in the people who had PL absence, and, in addition, it was found with a more tendency towards the male population [24]. Baker et al., in 1981, found that the absence of the PL and the variation of the FDS showed independence results [25]. Fortunately, the PL absence and the FDS variations do not affect the hand function. Mugalur et al. reported in 2015 no significant decline of the hand function with bilateral absence of the PL and FDS variations [15, 21]. In our study, we found a gender difference for the existence of the FDS variations but it did not support the effect of the dominance of the hands. We could not find any correlation between the PL absence and the FDS absence among all conditions.

Weaknesses of this study are considerable. Riyadh city harbors about 6 million people. Universities' student-volunteers do not represent the Riyadh city only but they are from scattered areas of the entire country. Since it was quite easy to access the universities that contained broad spectrum of the population so we chose this strategy to work on them. We recommend testing the PL and FDS absence among family members to determine the rate of inheritance. This will need a pedigree-based clinical analysis which may possibly explore the genetic background of the PL and FDS agenesis.

## 6. Conclusion

The prevalence of the bilateral absence of the Palmaris Longus (PL) and the Flexor Digitorum Superficialis (FDS) was observed as 15.1% and 14.8%, respectively, in the studied Saudi Arabian population. This was lower than the prevalence observed in the other Arabian nations, that is, 24.5%. The dominance of hands was not related to the tested variables found in PL and FDS agenesis. Variation of the FDS tendon absence was an independent entity for the PL absence.

## Figures and Tables

**Table 1:** General characterization of patients (*N* = 331).

	Overall
Gender	
Male	164 (49.5%)
Female	167 (50.5%)
Mean age (years)	23 (SD ± 5.3) years
Right hand dominance	294 (88.8%)
Left hand dominance	37 (11.2%)

**Table 2:** Characteristics of the Palmaris Longus (PL) muscle.

	Overall		
Present (both hands)	228 (68.9%)		
Absent (both hands)	50 (15.1%)		
Present (right hand)	27 (8.2%)		
Present (left hand)	26 (7.9)		

PL versus dominant hand	Rt. hand	Lt. hand	

Present (both hands)	200 (68.0%)	28 (75.6%)	*p value* = 0.788
Absent (both hands)	45 (15.3%)	5 (13.5%)	
Present (right hand)	25 (8.5%)	2 (5.4%)	
Present (left hand)	24 (8.1%)	2 (5.4%)	

PL versus gender	Male	Female	

Present (both hands)	99 (60.3%)	129 (77.2%)	*p value* = 0.001
Absent (both hands)	38 (23.1%)	12 (7.1%)	
Present (right hand)	16 (9.7%)	11 (6.5%)	
Present (left hand)	11 (6.7%)	15 (8.9%)	

*Significant p value ≤ 0.05*.

**Table 3:** Characteristics of the Flexor Digitorum Superficialis (FDS) muscle.

	Overall		
Present (both hands)	210 (63.4%)		
Absent (both hands)	49 (14.8%)		
Present (right hand)	40 (12.1%)		
Present (left hand)	32 (9.7)		

FDS versus dominant hand	Rt. hand	Lt. hand	

Present (both hands)	188 (63.9%)	22 (59.4%)	*p value* = 0.835
Absent (both hands)	44 (14.9%)	5 (13.5%)	
Present (right hand)	35 (11.9%)	5 (13.5%)	
Present (left hand)	27 (9.1%)	5 (13.5%)	

FDS versus gender	Male	Female	

Present (both hands)	118 (71.9%)	92 (55.0%)	*p value* = 0.001
Absent (both hands)	17 (10.3%)	32 (19.1%)	
Present (right hand)	21 (12.8%)	19 (11.3%)	
Present (left hand)	8 (4.8%)	24 (14.3%)	

*Significant p value* ≤ 0.05.

**Table 4:** Parameters of the Palmaris Longus (PL) versus Flexor Digitorum Superficialis (FDS) muscle.

Palmaris Longus	Flexor Digitorum Superficialis				
	Present (both hands)	Absent (both hands)	Present (right hand)	Present (left hand)	Total
Present (both hands)	142 (67.6%)	33 (67.3%)	27 (67.5%)	26 (81.2%)	228
Absent (both hands)	32 (15.2%)	11 (22.4%)	6 (15.0%)	1 (3.1%)	50
Present (right hand)	19 (9.0%)	2 (4.0%)	3 (7.5%)	3 (9.3%)	27
Present (left hand)	17 (8.09%)	3 (6.1%)	4 (10.0%)	2 (6.2%)	27
Total	210	49	40	32	**331**

*p*  *value* ≥ 0.595.
